# A role for B cells in organic dust induced lung inflammation

**DOI:** 10.1186/s12931-017-0703-x

**Published:** 2017-12-22

**Authors:** Jill A. Poole, Ted R. Mikuls, Michael J. Duryee, Kristi J. Warren, Todd A. Wyatt, Amy J. Nelson, Debra J. Romberger, William W. West, Geoffrey M. Thiele

**Affiliations:** 1 Pulmonary, Critical Care, Sleep & Allergy Division, Department of Internal Medicine, University of Nebraska Medical Center (UNMC), 985990 Nebraska Medical Center, Omaha, NE 68198-5990 USA; 2 Veterans Affairs Nebraska-Western Iowa Health Care System, Research Service, Omaha, NE USA; 3 Rheumatology Division, Department of Internal Medicine, UNMC, Omaha, NE USA; 4 Department of Environmental, Agricultural, and Occupational Health, UNMC, Omaha, NE USA; 5 Pathology and Microbiology Department, UNMC, Omaha, NE USA

**Keywords:** Lung, Inflammation, Autoantibody, Immunoglobulin, Organic dust, MAA adduct, ACPA

## Abstract

**Background:**

Agriculture organic dust exposures induce lung disease with lymphoid aggregates comprised of both T and B cells. The precise role of B cells in mediating lung inflammation is unknown, yet might be relevant given the emerging role of B cells in obstructive pulmonary disease and associated autoimmunity.

**Methods:**

Using an established animal model, C57BL/6 wild-type (WT) and B-cell receptor (BCR) knock-out (KO) mice were repetitively treated with intranasal inhalation of swine confinement organic dust extract (ODE) daily for 3 weeks and lavage fluid, lung tissues, and serum were collected.

**Results:**

ODE-induced neutrophil influx in lavage fluid was not reduced in BCR KO animals, but there was reduction in TNF-α, IL-6, CXCL1, and CXCL2 release. ODE-induced lymphoid aggregates failed to develop in BCR KO mice. There was a decrease in ODE-induced lung tissue CD11c^+^CD11b^+^ exudative macrophages and compensatory increase in CD8^+^ T cells in lavage fluid of BCR KO animals. Compared to saline, there was an expansion of conventional B2-, innate B1 (CD19^+^CD11b^+^CD5^+/−^)-, and memory (CD19^+^CD273^+/-^CD73^+/−^) B cells following ODE exposure in WT mice. Autoreactive responses including serum IgG anti-citrullinated protein antibody (ACPA) and anti-malondialdehyde-acetaldehyde (MAA) autoantibodies were increased in ODE treated WT mice as compared to saline control. B cells and serum immunoglobulins were not detected in BCR KO animals.

**Conclusions:**

Lung tissue staining for citrullinated and MAA modified proteins were increased in ODE-treated WT animals, but not BCR KO mice. These studies show that agriculture organic dust induced lung inflammation is dependent upon B cells, and dust exposure induces an autoreactive response.

**Electronic supplementary material:**

The online version of this article (10.1186/s12931-017-0703-x) contains supplementary material, which is available to authorized users.

## Background

Chronic bronchitis and chronic obstructive pulmonary disease (COPD) are common adverse respiratory health effects among persons exposed to agriculture organic dust work environments [1, 2]. Agriculture organic dusts are complex, comprised of particulates and enriched with a diversity and abundance of gram negative and gram positive microbial components [3–5]. These exposures elicit innate immune responses through activation of Toll-like receptors (TLR; i.e. TLR2, TLR4, TLR9), scavenger receptors, and myeloid differentiation factor 88 (MyD88) signaling pathways [6–9]. Human and animal studies have also described roles for activated lung macrophages, CD4^+^ T cells, and a Th1/Th17 lung microenvironment as related to agriculture organic dust exposures [10–14]. Earlier animal studies demonstrated that repetitive organic dust extract (ODE) exposures induce discrete peribronchiolar and lung parenchymal lymphoid aggregates comprised of both T and B cells [15]. However, the precise role of B cells in mediating agriculture organic dust-induced lung inflammation has not been thoroughly investigated. This is increasingly relevant given the emerging role that B cells are understood to play in COPD and COPD-associated autoimmunity [16], pathways that could potentially be targeted therapeutically.

Increased numbers of large and small airway associated B cells correlate with COPD severity [17], but the role of these B cells is controversial. B cell responses can be protective against microbial infection through production of an antibody response to promote pathogen clearance, but B cells might also be harmful by directing an autoantibody response against lung tissue antigens [18]. It has long been recognized that agricultural workers exposed to a variety of environmental agents mount antigen-driven, humoral responses. This has been most evident in hypersensitivity pneumonitis whereby the inciting antigen is due to microorganisms found in hay or grain [19]. More recently, cigarette smoke exposure resulting in lung inflammation and oxidative stress has been shown to induce autoantibodies such as anti-citrullinated protein antibody (ACPA) that are implicated in rheumatoid arthritis (RA) as well as autoantibodies to heat shock protein and oxidized lipoproteins [20, 21]. Moreover, malondialdehyde-acetaldehyde (MAA) protein adducts are a general product of oxidative stress that are expressed in a variety of inflammatory disease states including atherosclerosis [22, 23], alcoholic liver disease [24], rheumatoid arthritis [25], and alcohol/smoking-associated lung inflammation [26]. This is important because MAA adducts are highly immunogenic and lead to robust adaptive immune responses to both the MAA epitope and carrier proteins. It is not known whether agriculture organic dust-induced lung inflammation induces autoreactive antibodies to endogenous proteins including those characterized by the presence of MAA adducts or citrullinated residues. The relationship of agricultural dust exposures with B cell mediated lung inflammation and autoimmunity development may have added relevance as farming occupations as well as other pulmonary inhalant have been associated with autoimmune diseases including RA [27–31].

In this study we hypothesize that B cells are important in mediating organic dust-induced lung disease and immunoglobulin responses, including the expression of autoantibodies. To test this hypothesis, organic dust-induced lung inflammatory consequences, lung B cell subset responses, and systemic IgM, IgA, IgG, and IgE response in wild-type (WT) and B-cell receptor (BCR) knock out (KO) mice were investigated. Additionally, we sought to determine whether autoantibodies to endogenous products associated with autoimmune disease pathogenesis including ACPA and anti-MAA antibodies were induced. Our studies show that repetitive agriculture organic dust-induced lung inflammation is dependent upon B cells, and that this dust exposure induces autoantibody responses.

## Methods

### Organic dust extract

Aqueous organic dust extract (ODE) was prepared as previously described [15]. Briefly, settled dust was collected from horizontal surfaces (~1 m above floor level) of swine confinement feeding facilities (~400-600 animals) located in Colfax County, Nebraska (population density approximates 25 people per square mile). Dust (1 g) was incubated in sterile Hank’s Balanced Salt Solution (10 mL; Mediatech, Manassas, VA) at room temperature for 1 h and centrifuged for 30 min at 2850 × *g*. The supernatant was removed and then spun again for 30 min at 2850 g. The final supernate was filter-sterilized (0.22 μm) to remove microorganisms and coarse particles. Endotoxin concentrations in 100% ODE ranged from 1200 to 1400 EU/mL as determined using the limulus amebocyte lysate assay (Lonza, Walkersville, MD). Muramic acid levels (bacterial cell wall peptidoglycans) were previously determined by mass spectrometry to be approximately 70 ng/mg [32]. Stock ODE was batched prepared, stored at −20 °C, and aliquots were diluted for each experiment to a final concentration (vol/vol) of 12.5% for animal studies in sterile phosphate buffered saline (PBS; pH 7.4; diluent). ODE 12.5% has been previously shown to elicit optimal experimental outcomes in mice and is well tolerated [15].

### Animals

All animal procedures were approved by the University of Nebraska Medical Center Institutional Animal Care and Use Committee and were in accordance with the NIH guidelines for the use of rodents. WT C57BL/6 mice and B cell receptor KO (Strain B6.129S2-Igh-6^tm1Cgn^/J) on C57BL/6 background were purchased from The Jackson Laboratory (Bar Harbor, ME). Male mice, between 6 and 10 weeks of age, were used for all studies because the occupational animal confinement facility industry is predominately male. All mice had ad libitum access to standard rodent chow and filtered water through the course of the studies.

### Animal exposure model

An established intranasal inhalation repetitive exposure animal model was utilized whereby mice were lightly sedated under isoflurane and received treatment with either 50 μL of sterile saline (PBS) or 12.5% ODE daily for 3 weeks [15]. Animals were euthanized 5 h following the final exposure for experimental endpoint quantification. No respiratory distress, signs of stress, or weight loss throughout the treatment period was observed.

### Bronchoalveolar lavage fluid and lung tissue homogenate cell analysis

Immediately after the animals were euthanized, bronchoalveolar lavage fluid (BALF) was accumulated using 3 × 1 mL PBS. Total cell numbers from the combined recovered lavage were enumerated and differential cell counts determined from cytospin-prepared slides (cytopro cytocentrifuge, ELITech Group, Logan, UT) stained with DiffQuick (Siemens, Newark, DE). From cell-free supernate of the first lavage fraction, tumor necrosis factor-alpha (TNF-α), interleukin-6 (IL-6) and murine neutrophil chemokines, CXCL1 and CXLC4, were quantitated by ELISA (R&D Systems, Minneapolis, MN) because they have been implicated in agriculture exposure-induced lung inflammation [9, 10, 15]. IL-17 and total hyaluronan, B cell chemoattractants [33–36], were also characterized in total lung homogenates. Briefly, lung homogenates were prepared by homogenizing ½ lung samples in 500 μl of sterile PBS as previously described [12]. Levels were determined according to manufacturer’s instruction using an enzyme-linked immunosorbent assay kit for IL-17A (Abcam, Cambridge, MA) and hyaluronic acid (Echelon Biosciences, Inc., Salt Lake City, UT) with sensitivities of 6.25 pg/ml and 12.5 ng/ml, respectively.

### Histopathology

Following lung lavage, whole lungs were excised and slowly inflated (15 cm H_2_O pressure) with 10% formalin (Sigma, St. Louis, MO) for 24 h to preserve pulmonary architecture as previously described [15]. Fixed lungs were processed, embedded in paraffin, and entire lung sections were cut (4-5 μm) and stained with hematoxylin and eosin (H&E). Each slide was entirely reviewed at scanning magnifications (2X, 4X, and 10X objectives; Nikon Eclipse Model E600 microscope, Nikon, Tokyo, Japan) and semi-quantitatively assessed for the degree and distribution of lung inflammation by a pathologist (W.W.W.), blinded to the treatment conditions, utilizing a previously published scoring system [15]. This scoring system evaluates the spectrum of inflammatory changes for: 1) alveolar compartment inflammation, 2) bronchiolar compartment inflammation, and 3) intrapulmonary lymphoid aggregates and is compared to a standard set of photomicrographs. The bronchiolar compartment consists of inflammatory infiltrates in both the peri-bronchiolar and per-vascular space. Each parameter was independently assigned a value from 0 to 3, with a higher score indicating greater inflammatory changes in the lung.

### Cell staining and flow cytometry

In separate studies following collection of lung lavage fluid cells, the chest cavity was opened and the right ventricle was infused with 10 mL of sterile PBS with heparin to remove blood from the pulmonary vasculature. Half of the lung was harvested and subjected to an automated dissociation procedure using a gentleMACS Dissociator instrument (Miltenyi Biotech, Auburn, CA) in a solution containing collagenase type I (324 U/mL; Fisher, Pittsburgh, PA), bovine DNAse (75 U/mL), porcine heparin (25 U/mL), and PBS with Ca^2+^ and Mg^2+^. Cell solution was passed through nylon mesh (40 μM; Fisher) to remove any large fragments and red blood cells were lysed using a 0.84% (*w*/*v*) ammonium chloride treatment (5 min at 4 °C). All reagents not specifically stated were obtained from Sigma. The cells were then resuspended in PBS, and lung cells were isolated by density gradient centrifugation over Ficoll-Paque PLUS (GE Healthcare, Uppsala, Sweden), enumerated, and reported as “total lung cells.” Viability of the final cell preparation was assessed by trypan blue exclusion and a LIVE/DEAD fixable Violet Dead Cell Stain Kit (Life Technologies, Carlsbad, CA). Ultimately, <1% of gated cells were not viable, with no difference noted between the saline and ODE-treated groups (data not shown).

BALF and lung cells from each animal were incubated with CD16/32 (Fc Block, BD Biosciences, San Jose, CA) to minimize non-specific antibody staining, and then stained with monoclonal antibodies (mAb) directed against rat anti-mouse CD45 (clone: 30-F11), CD11b (clone: M1/70), Ly6G (clone RB6-8C5), CD4 (clone RM 4-5), CD8 (clone: 53-6.7), and hamster anti-mouse CD3 (clone: 145-2C11) from BD Biosciences (San Jose, California), rat anti-mouse CD19 (clone: eBio1D3) and CD11c (clone: N418) from eBiosciences (Waltham, MA), and rat anti-mouse CD5 (clone: 53-7.3), CD273 (clone: TY25), CD73 (clone: TY/11.8) from Biolegend (San Diego, CA). Parallel cell preparations were treated with appropriate isotype control antibodies, and compensation was performed with antibody capture beads (BD Biosciences) stained separately with each individual mAb used in test samples.

The gating strategy for CD11c^+^CD11b^lo^ alveolar macrophages, CD11c^+^CD11^hi^ exudate macrophages, Ly6G^+^ neutrophils, CD3^+^CD4^+^ and CD3^+^CD8^+^ T cells were previously reported [12, 13]. B1, B2 B and memory B cell subpopulations were identified according to published reports by others: B1 B cells (CD19^+^CD11b^+^), B1a B cells (CD19^+^CD11b^+^CD5^+^), B2 B cells (CD19^+^CD11b^−^) [37], and the spectrum of murine memory B cell subsets identified by CD273 and CD73 expression of CD19^+^ B cells [38]. The percentage of all respective cell populations were determined from CD45^+^ lung leukocytes after excluding debris and doublets. This percentage was multiplied by the respective total BALF or lung cell numbers to determine specific cell population numbers for each animal.

### Preparation of modified proteins

For these experiments, aqueous human albumin (Alb) purchased from Talecris Biotherapeutics, Inc., Research Triangle Park, NC was incubated with 1 mM Acetaldehyde (AA) obtained from Aldrich Chemical Co. (Milwaukee, WI, USA) and 2 mM Malondialdehyde (MDA) obtained as the sodium salt (MDA–Na) by treatment of tetramethoxypropane (Aldrich Chemical Co.) with NaOH, according to the method of Iwata and Kikugawa [39] to form the MAA adduct. Alternatively, a quantitative ELISA for MAA adducted protein was performed on mouse lung homogenates as previously described [26].

### Serum

Whole blood was collected from mice at the time of euthanasia from the axillary artery. Blood (400 μL) was placed in BD Microtainer Tubes (Becton, Dickinson and Company, Franklin Lakes, NJ), centrifuged for 2 min at 6000 × g and supernatant collected. Serum IgM, IgA, IgG and IgE were quantified according to manufacturer’s instruction using a Quantikine enzyme-linked immunosorbent assay kit (Affymetrix eBioscience, Santa Clara, CA).

Serum anti-MAA antibodies (IgG) were quantified as previously described [25]. Aqueous human albumin was adducted with malondialdehyde and acetaldehyde (2:1). ELISA plates were coated with 2 μg/well of MAA-albumin or albumin. Additional wells were coated with known concentrations of murine IgG isotype standards from which relative antibody concentration were extrapolated. Plates were incubated overnight at 4 °C, washed and blocked with 3% bovine serum albumin, and incubated with each mouse serum at a 1:1000 dilution. Following incubation at 37 °C for 1 h, a secondary horseradish peroxidase goat anti-mouse antibody specific for IgG Fcγ fragment specific (Jackson ImmunoResearch, West Grove, PA) was added. Plates were developed using TMB substrate, absorbance determined at 450 nm using an Epoch Plate reader (BioTek, Winooski, VT) and analyzed using Gene 5 Software (BioTek). Data are presented as arbitrary units (AU) of the antibody detected in the assay as this reflects the amount of antibody present in a sample relative to a standard curve.

Serum anti-citrullinated peptide antigen (ACPA) was determined using a modification of the second-generation anti-CCP antibody ELISA (Diastat; Axis-Shield Diagnostics). As previously described [40], mouse serum was incubated on the anti-CCP plate, washed, and then a HRP goat anti-mouse antibody specific for IgG Fcγ fragment specific (Jackson ImmunoResearch) detection antibody replaced the anti-human IgG antibody from the kit.

### Immunohistochemistry

Formalin-fixed, paraffin-embedded lung tissue sections of 4- to5-μm-thickness lung tissue were deparaffinized and antigen unmasking was performed using antigen retrieval techniques as per Bethyl Laboratories protocol and buffers (Montgomery, TX). The rabbit anti-MAA antibody and rabbit IgG were first labeled with a Zenon™ Alexa Fluor™ 405 rabbit IgG labeling kit prior to incubation with the sample (Life Technologies Corporation, Eugene, Oregon). Slides were blocked with 2% goat serum and sections stained for the presence of MAA adducted proteins using an affinity purified MAA-specific rabbit polyclonal antibody, anti-peptidyl-citrulline, clone F95 antibody (EMD Millipore Corporation, Temecula, CA) and macrophages using a ALEXA FLUOR™ 594 Conjugated Rabbit Anti-CD68 Polyclonal Antibody (Bioss Antibodies, Woburn, MA). The anti-citrulline antibody was detected using a Cy™3-conjugated AffiniPure F(ab’)2 Fragment Goat Anti-Mouse IgM, μ Chain Specific (minimal cross-reaction to Human, Bovine, and Horse Serum Proteins) (Jackson ImmunoResearch). Isotype controls included a Zenon™ labeled rabbit IgG, mouse IgM + the Cy3 goat anti-mouse secondary, and ALEXA FLUOR® 594 Conjugated Rabbit IgG (Bioss Antibodies). Slides containing the primary antibodies were incubated overnight at 4 °C in a humidified chamber. The next day slides were washed in PBS and incubated with the secondary antibody for 1 h, washed, mounted, and imaged using a Zeiss 510 Meta Confocal Laser Scanning Microscope. All images were analyzed using ZEN 2012 software (Zeiss). Image quantification was done using Image J software (National Institutes of Health) and represented as mean (± standard error of mean [SEM] pixel density).

### Statistical methods

Data are presented as the mean ± standard error of mean (SEM). To detect significant changes between groups, a one-way analysis of variance (ANOVA) was utilized and a post hoc test (Tukey/LSD) or nonparametric Mann-Whitney test was performed to account for multiple comparisons if the *p* value was <0.05. All statistical analysis were performed using GraphPad Prism software (La Jolla, CA) and statistical significance accepted at *p* < 0.05.

## Results

### Airway inflammatory cytokine/chemokine response, but not cellular influx, is reduced in BCR KO mice following repetitive ODE treatments

**Fig. 1:**
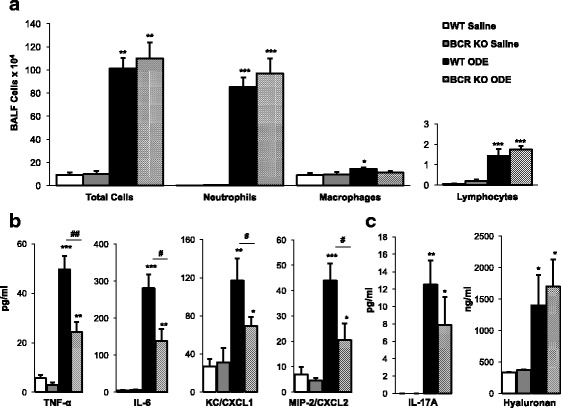
Airway inflammatory cell influx and mediator response following repetitive ODE exposure in B-cell receptor (BCR) knockout mice (KO) mice. Mice were intranasally treated with saline or organic dust extract (ODE) daily for 3 weeks and bronchoalveolar lavage fluid (BALF) was collected 5 h following final exposure. Bar graphs of means with standard error bars of **a** total cells and cell differentials and **b** cytokine/chemokine levels quantitated in BALF are shown. **c** Mean levels with standard error bars of B-cell chemotactic mediators IL-17A and hyaluronan quantitated in lung tissue homogenates are shown. There is no difference in ODE-induced cellular influx, IL-17A, or hyaluronan between WT and KO mice. ODE-induced TNF-α, IL-6, murine neutrophil chemoattractants (CXCL1 and CXCL2) response were reduced in BCR KO animals. *N* = 6 mice/treatment group from 2 independent experiments. Statistical significance (*p < 0.05, **p < 0.01, ***p < 0.001) vs. matched saline. Significant differences between WT and BCR KO denoted by line (#*p* < 0.05, ##*p* < 0.01)

Consistent with previous reports [15], intranasal inhalation of 12.5% ODE daily for 3 weeks resulted in an increased influx of neutrophils, macrophages and lymphocytes and increases in TNF-α, IL-6, CXCL1 and CXCL2 concentrations in BALF from WT mice (Fig. 1a-b). Repetitive ODE treatments resulted in similar increases in total airway cells, neutrophils and lymphocytes in BCR KO mice as compared to WT animals. Mean ± SEM (pg/ml) BALF concentrations of ODE-induced TNF-α (49.7 ± 5.5 vs. 24.4 ± 4.0; *p* = 0.0025), IL-6 (281.1 ± 36.9 vs. 138.4 ± 31.6; *p* = 0.015), CXCL (116.9 ± 23.5 vs. 69.5 ± 9.0; *p* = 0.038), and CXCL2 (43.94 ± 6.7 vs.20.4 ± 6.5; *p* = 0.035) were significantly lower in BCR KO mice when compared to WT animals (Fig. 1b). IL-17A and hyaluronan are B-cell chemoattractants [33–36] and repetitive ODE treatment resulted in increased IL-17A and hyaluronan concentration in lung tissue homogenates from WT and BCR KO animals as compared to saline (Fig. 1c). Levels of IL-17A and hyaluronan in BALF were below the lower limit of detection in all treatment groups (data not shown).

### B cells are essential for the formation of lymphoid aggregates following ODE treatments

**Fig. 2:**
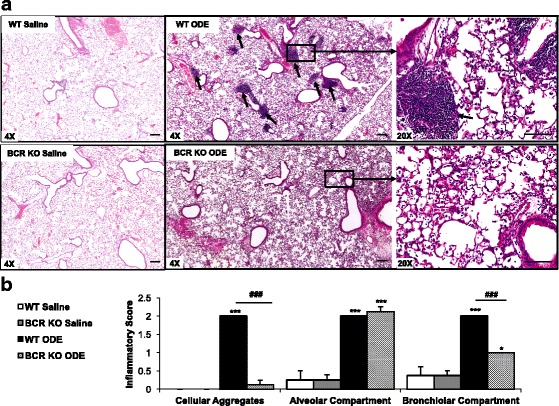
B cells are essential for the formation of ODE-induced peribronchiolar cellular aggregates, but do not explain alveolar compartment inflammation. WT and BCR KO mice were repetitively exposed to saline or ODE for 3 weeks. Whole lung sections (4-5-μm) were stained with hematoxylin and eosin. **a** A representative lung section from each treatment group is shown at 4X magnification. A 20X magnification image of boxed area depicted in 4X image of WT and BCR KO + ODE is shown on far right panel. Short arrows indicate cellular aggregates. **b** Bar graph depicts mean with standard error bars of the semi-quantitative degree and distribution of lung cellular aggregates, alveolar inflammation, and bronchiolar inflammation with *N* = 4-6 mice/group. Line scale is 100 μm. Statistical difference (*p < 0.05, ***p < 0.01) vs. matched saline. ###*p* < 0.001 is significant difference between WT and BCR KO animals

Repetitive ODE exposure results in lung pathology marked by an increase in lymphoid aggregates, alveolar compartment inflammation, and bronchiolar compartment inflammation [15]. By microscopic review, there was a striking reduction in the development of lymphoid aggregates and peribronchiolar inflammation in BCR KO mice treated repetitively with ODE as compared to ODE-treated WT animals (Fig. 2a). By semi-quantitative assessment, the frequency and distribution of ODE-induced lymphoid aggregates and bronchiolar compartment inflammation were significantly reduced in BCR KO mice (Fig. 2b). There was no difference in the semi-quantitatively graded distribution of lung alveolar inflammation between ODE-treated WT and BCR KO animals. Collectively, these studies indicate that B cells are a critical component of ODE-induced lung lymphoid aggregates and peribronchiolar histopathology.

### Impact of ODE-induced cellular influx in lavage fluid and lung tissue compartments in BCR KO animals assessed by flow cytometry

**Fig. 3:**
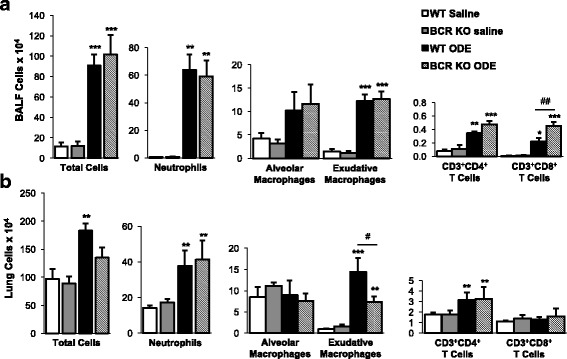
Impact of ODE-induced cellular influx in lavage fluid and lung tissue compartments in BCR KO animals. WT and BCR KO mice were repetitively exposed to saline or ODE daily for 3 weeks whereupon **panel a** BALF and **panel b** dissociated lung tissue cells were processed and analyzed by flow cytometry. BALF total cells were determined by hemocytometer. Total lung cells represent ½ whole lung tissue cells following density gradient separation as determined by hemocytometer. Numbers of neutrophils (Ly6G^+^), alveolar macrophages (CD11c^hi^CD11b^lo^), exudative macrophages (CD11c^hi^CD11b^hi^), CD3^+^CD4^+^ T cells, and CD3^+^CD8^+^ T cells were calculated by multiplying the percentage of cells in respective gate (% of CD45^+^ cells, as analyzed by FACS) multiplied by respective total cells for each mouse. Bar graphs depict means with standard error bars. N = 6-8 mice/group from 2 independent studies. Statistical significance (*p < 0.05, **p < 0.01, ***p < 0.001) versus saline. Line denotes significant difference (#*p* < 0.01, ##*p* < 0.01) between WT and KO

To further understand the impact of B cells on modulating ODE-induced inflammatory cellular phenotypes in the airway lavage fluid vs. lung tissue compartment, separate experimental animal studies were conducted by subjecting the same animal lavage fluid cells and lung tissues cells to flow cytometry analysis. In BALF, there was no difference in numbers of total cells, neutrophils (Ly6G^+^), alveolar macrophages (CD11c^hi^CD11b^lo^), or exudative macrophages (CD11c^hi^CD11b^hi^) in ODE-treated WT vs. BCR KO mice (Fig. 3a). In contrast, there was an increase in ODE-induced CD3^+^CD8^+^ T cells in the BCR KO animals as compared to WT animals. In the lung tissue compartment, there was no difference between ODE-treated WT and BCR KO mice in regards to the numbers of neutrophils alveolar macrophages, or CD3^+^CD4^+^ T cells (Fig. 3b). However, there was a reduction in ODE-induced exudative (or activated) macrophages in BCR KO animals as compared to WT mice (Fig. 3b). There was no significant increase in lung CD3^+^CD8^+^ T cells with ODE treatment compared to saline in either WT or BCR KO mice.

### B1 and B2 B cell subpopulations are increased following repetitive ODE exposure

**Fig. 4:**
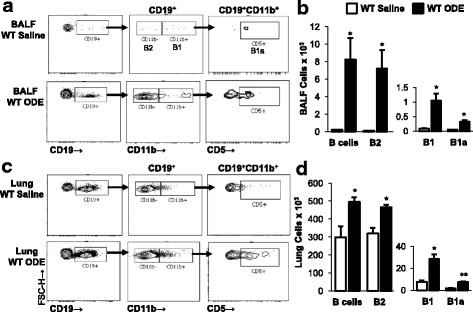
Repetitive ODE exposure induces airway and lung compartment B1 and B2 populations. WT and BCR KO mice were repetitively exposed to saline or ODE daily for 3 weeks whereupon BALF and dissociated tissue cells were processed and analyzed by flow cytometry. Lymphocytes identified by CD45^+^ leukocytes excluding debris and characteristic FSC and SSC properties of lymphocytes followed by staining for CD19, CD11b, and CD5. A representative contour plot of **a** BALF cells and **c** lung tissue cells demonstrating gating strategy for CD19^+^ B cells, B2 B cells (CD19^+^CD11b^−^), B1 B cells (CD19^+^CD11b^+^), and B1a B cells (CD19^+^CD11b^+^CD5^+^) from WT saline and ODE treated animals is shown. Numbers of B, B2, B1, and B1a cells were calculated by multiplying the percentage of cells in respective gate (% of CD45+ cells, as analyzed by FACS) multiplied by respective total cells (see Fig. 3) for each mouse. Bar graphs depict means with standard error bars of **b** BALF and **d** lung tissue cells of 6 mice/group. There were no B cells in the BCR KO mice. Statistical difference (*p < 0.05; **p < 0.01) versus saline

To the best of our knowledge, there have been no prior studies investigating the role of ODE in regulating various B cell subpopulations. B2 B cells, which are hallmark effectors of the adaptive immune responses and B1 B cells, which are considered the innate immune component providing source of natural/self-reactive antibodies, were determined in same animal lavage fluid cells and lung tissue cells in order to provide information on airway and lung tissue response to ODE exposure. In WT mice, there was a robust increase in the number of B2 B cells in both BALF (Fig. 4a) and lung tissues (Fig. 4b) after repetitive ODE exposures as compared to saline exposure. There were also increases in B1 and B1a B cells, but to a lesser magnitude, following ODE exposure in both BALF and lung tissue (Fig. 4a-b). There were no detectable B cells in the saline and ODE treated BCR KO mice (data not shown).

### Memory B cell subpopulations are increased following repetitive ODE exposure

**Fig. 5:**
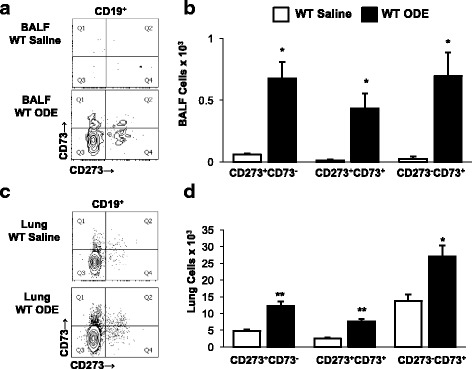
Repetitive ODE treatments induce memory B cell subpopulations. WT and BCR KO mice were repetitively exposed to saline or ODE daily for 3 weeks whereupon BALF and dissociated tissue cells were processed and analyzed by flow cytometry. Lymphocytes identified by CD45^+^ leukocytes excluding debris and characteristic FSC and SSC properties of lymphocytes followed by staining for CD19, CD73, and CD273. A representative contour plot of **a** BALF cells and **c** lung tissue cells demonstrating gating strategy for memory B cell populations from WT saline and ODE treated animals is shown. Numbers of memory B cells were calculated by multiplying the percentage of cells in respective gate (% of CD45+ cells, as analyzed by FACS) multiplied by respective total cells (see Fig. 3) for each mouse. Bar graphs depict means with standard error bars of **b** BALF and **d** lung tissue cells of 6 mice/group. There were no B cells in the BCR KO mice. Statistical difference (*p < 0.05; **p < 0.01) versus saline

To explore whether ODE exposure also impacts the heterogeneity of memory B cells subsets, B cells from BALF and lung tissues were investigated for expression of CD73 and CD273 (also known as PD-L2). Although the magnitude of response was not robust, there were significant increases in all memory B cell subsets following ODE treatment in both lavage fluid and lung tissues (Fig. 5). There were no memory B cells detected in the saline- or ODE-treated BCR KO mice (data not shown).

### Repetitive ODE treatment induces total serum IgG and IgE levels that are abrogated in BCR KO mice

As B cells function to produce antibodies, the serum immunoglobulin (Ig) responses following repetitive ODE exposure were quantified. ODE treatment resulted in significant increases in serum IgG and to a lesser degree IgE, but not IgM or IgA, levels in WT mice (Table 1). As anticipated, there was no detection of IgA, IgM, IgG and IgE in saline and ODE-treated BCR KO animals (data not shown).

**Table 1:** Serum immunoglobulin levels (μg/mL) in wild-type mice treated with saline and organic dust extract (ODE) for 3 weeks. Immunoglobulins not detected in saline- or ODE-treated B cell receptor knockout animals

	IgG	IgM	IgA	IgE
Saline	2797 (623.2)	394.5 (112.1)	17.1 (1.02)	0.094 (0.052)
ODE	7628 (791.3)*	278.8 (30.5)	19.58 (0.36)	1.28 (0.23)*

Mean (SEM) shown. Statistical Significance denoted as *p < 0.05 vs. Saline treatment. N = minimum of 4 mice/group

### Repetitive inhalation of ODE increases autoreactive antibodies to MAA and citrullinated proteins

**Fig. 6:**
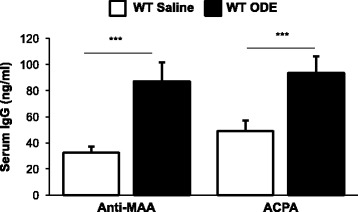
Repetitive ODE exposure induces anti-MAA and anti-ACPAP IgG antibodies. WT were intranasally treated with saline or ODE daily for 3 weeks. Autoreactive IgG antibodies to malondialdehyde-acetaldehyde (MAA) and citrullinated protein antibody (ACPA) were quantified in serum by ELISA. Graph depicts mean with standard error bars of *N* = 11-13 mice/group from a minimum of 3 independent studies. There were no autoantibodies detected in the BCR KO mice. Statistical significance between saline and ODE (***p < 0.001)

In the next set of experiments, we sought to determine whether there was a specific ODE-mediated autoantibody response to endogenous proteins associated with other inflammatory diseases and autoimmunity, specifically anti-MAA and anti-citrullinated protein antibody (ACPA). First, to determine whether ODE-induced lung inflammation resulted in the production ACPA and anti-MAA antibodies, sera from saline- and ODE-treated mice were analyzed by ELISA. There were significant increases in anti-MAA and anti-CCP antibody levels in WT animals repetitively treated with ODE as compared to saline control (Fig. 6). Isotype control staining images are shown in Additional file 1: Figure S1. Anti-MAA and anti-CCP antibodies were not detected in the BALF or lung tissues of the animals (data not shown). Furthermore, these antibodies were not detected in BCR KO mice. These data demonstrate that ODE exposure induces a systemic autoantibody response to endogenous, pathogen disease state antigens.

### Repetitive ODE exposure induces CIT and MAA modified proteins in the lung of WT animals

**Fig. 7:**
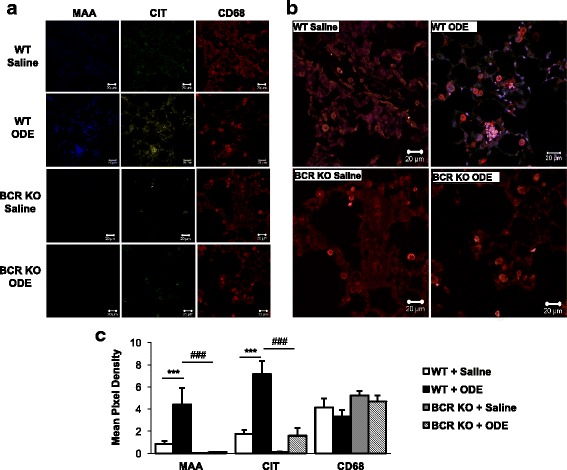
ODE exposure-induced MAA and citrullinated modified proteins in the lung tissue of WT mice is diminished in BCR KO animals. Confocal images of lung tissue from WT and BCR KO mice treated repetitively with saline or ODE were stained for malondialdehyde-acetaldehyde (MAA) adducted protein, citrulline (CIT), and CD68 (macrophage marker). Sections were stained, mounted in fluoromount-G, and subjected to confocal microscopy using a Zeiss 710 Meta confocal laser-scanning microscope at 40× magnification. Images were analyzed using Zen 2012 software (Zeiss). **a** Individual staining of WT-Saline or ODE and BCR-KO Saline or ODE for anti-MAA (blue), anti-Citrulline (green), and anti-CD68 (red). **b** Merged images show co-localization of MAA, CIT, and CD68 on lung tissue from treatment groups. **c** Mean pixel density of lung tissue stained for MAA, CIT, or CD68. ***p < 0.001 significantly increased compared to saline and ###*p* < 0.001 significantly decreased compared to BCR-KO + ODE. *N* = 5 separate murine lung section/group

In these studies, lung tissue from WT and BCR KO mice repetitively exposed to saline or ODE were stained and analyzed for the presence of MAA modified epitopes and citrullinated (CIT) antigens. By confocal microscopy, both CIT and MAA modified proteins detected predominantly in ODE-treated WT animals as compared to saline exposed animals, which was predominately in macrophages (CD68^+^ lung cells) and co-localized (Fig. 7a-b). This response was abrogated in the BCR KO animals. Quantification of staining demonstrated significant increase in MAA and CIT intensity in ODE treated WT animals as compared to saline treatment as well as ODE treated BCR KO mice (Fig. 7b). Furthermore, quantitative ELISA demonstrated significantly more MAA adducted protein in lung homogenates from ODE treated WT mice (24.5 ± 2.5 ng/ml) than in saline treated WT mice (12.7 ± 1.3 ng/ml) (*p* < 0.05, *N* = 8). There was no increase in MAA following ODE treatment in BCR KO mice (saline: 15.9 ± 2.3 ng/ml vs. ODE: 15.7 ± 1.4 ng/ml, *p* > 0.05, N = 8). Collectively, these studies demonstrate the presence and co-localization of both citrullinated and MAA modified proteins in the lung. In addition to mediating specific antibody responses to these epitopes, these results suggest that B cells also appear to play a role in the expression of these modified proteins in response to inhalant ODE exposure.

## Discussion

In this study, we demonstrated that B cells are central to the development of lung lymphoid aggregates following organic dust exposures and that these exposures induce B2, B1, and memory B cell subpopulations with a systemic immunoglobulin response. Moreover, we are the first to describe that organic dust-induced lung inflammation induces an autoantibody response of ACPA and anti-MAA antibody positivity with detection of both citrullinated and MAA modified proteins in lung tissue, which was abrogated in BCR KO animals. Taken together, these data suggest that B cells comprising ODE-induced lymphoid aggregates could be playing an important role in perpetuating agriculture-related lung disease, and that part of the inflammatory response is associated with the production of endogenous proteins resulting in self-reactivity. It is also possible that these autoantibodies could potentially serve as biomarkers of disease and/or subsequent generation of systemic autoimmune responses in exposed workers.

Despite reductions in hypersensitivity pneumonitis and allergic disorders in farmers, studies continue to show that respiratory complaints remain highly prevalent and airflow obstruction/COPD is common, even amongst non-smokers [1, 41, 42]. Of the various farming environments, livestock workers demonstrate the highest prevalence of respiratory symptoms, chronic bronchitis, and COPD [1, 2, 42]. With limitations in current therapeutic approaches for this population, a better understanding of the pathogenesis of disease could lead to alternative approaches. With the emerging interest in B cells and COPD pathogenesis and autoimmunity [16], we investigated the role of B cells and ODE exposure-induced lung disease. As in human COPD where there is an expansion of B cells, memory B cells, and B cell-rich lymphoid follicles [16], we found that B cells were important in explaining repetitive ODE exposure-induced lung pathogenesis. In the absence of B cells, there were decreased TNF-α, IL-6, and CXCL2 concentrations in lavage fluid, a failure to develop lymphoid aggregates with reductions being most prominent in the bronchiolar as opposed to alveolar compartment, and decreased lung exudative (CD11c^+^CD11b^+^) macrophages following ODE exposure. However, there was no reduction in ODE-induced lung neutrophil or T lymphocyte influx. As prior studies demonstrated that macrophages play a role in regulating airway inflammation following ODE exposure [13] and that lymphoid aggregates are comprised of T cell, B cell and macrophages [15], it is possible that the failure to form lymphoid aggregates explains the reduced inflammatory consequences. Furthermore, B cells as well as macrophages are sources of TNF-α, IL-6, and CXCL2 [13, 34]. This current study might also suggest that targeting B cells (e.g. anti-CD20 therapy) could reduce disease in affected agriculture workers. Moreover, our studies demonstrated an expansion of the conventional B2 B cells as well as increases in B1 and memory B cells.

B1- and B2- B cells secrete immunoglobulin, but murine B1 subsets (CD11b^+^CD5^+^ and CD11b^+^CD5^−^) are implicated in innate defense against mucosal pathogens and autoimmunity development [34]. ODE exposure increased B1 B cells, albeit the relative numbers of these cells were low. We speculate that these cells might be important in the autoreactive response to MAA and citrullinated modified proteins demonstrated following lung inflammation induced by ODE. Despite the increasing evidence of B1 B cell importance in mice, an equivalent subset in humans has not been definitively characterized due to difference between murine and human CD5 expression on B cells [34]. However, there are elevated fractions of human B1 B cells that express CD11b in patients with lupus [43]. To the best of our knowledge, there are no studies investigating the role of B1 B cells and COPD. Memory B cells (CD20^+^CD27^+^) are increased in the serum of patients with COPD [44] and plasma cells are found in COPD lung [45]. Memory B cells are a heterogeneous population [38], and our studies found expansion of these cells following ODE exposures. These current studies would support phenotyping B cell populations in agriculture workers to determine associations with disease.

There was an increase in total serum IgG levels in animals repetitively exposed to ODE, and to a much lesser degree, there was also an increase in IgE levels. The significance of the small degree of serum IgE increase is not known as there were no corresponding findings of an influx of eosinophils into the BALF or lung tissues following ODE exposure. We did find that repetitive ODE exposure increased specific IgG responses to MAA and citrullinated modified proteins. ACPAs serve as informative biomarkers of RA, and although they appear unable to induce arthritis alone, they can enhance the development of arthritis in mice when mild synovitis is present. To date, pathogenic consequences of anti-MAA antibodies have not been described. We suggest that these autoantibodies might serve as biomarkers of inflammatory disease in agriculture exposed persons, and future studies in humans are warranted. These antigens were detected in ODE-exposed lung tissue of WT mice. Historically, specific serum immunoglobulin responses to microorganisms in hay or grain attributable to agriculture work have been well described as in hypersensitivity pneumonitis [19]. Recently, it was demonstrated that occupational exposure to livestock (i.e. swine, poultry, and cattle) was associated with a trend (*p* = 0.1) toward increased levels of anti-ganglioside autoantibodies [46]. These authors had investigated anti-ganglioside autoantibody association because there was a higher prevalence of self-reported symptoms of peripheral neuropathy in the Agricultural Health Study farmers who worked with animals [47]. Interestingly, the farming occupation is associated with an increased prevalence of RA morbidity and mortality [28, 29].

There is an emergence of autoantibodies that recognize post-translational protein modifications that form under conditions of oxidative stress and chronic inflammation [48]. Serum ACPA is a prominent example whereby antibodies are directed against a wide array of citrullinated proteins and commonly associated with RA [48] or B-cell chemotactic factors. ACPA can present years prior to the development of autoimmune disease; and moreover, airborne stimuli including tobacco smoking and air pollution (industrial PM2.5 and SO_2_ emissions) have been linked to ACPA positivity [21, 49]. Less extensively studied are self-protein modifications through malondialdehyde (MDA) or MAA adduction. A large number of self-proteins are modified by MDA under inflammatory conditions and acetaldehyde can further react with MDA to form immunogenic MAA protein adducts [50]. It has been proposed that in the settings of chronic inflammation, citrullination and MDA/MAA-modifications could simultaneously arise [51]. We focused on the formation of MAA protein modifications because it has been shown that an increase of anti-MAA reactivity was associated with ACPA positivity without any direct cross-reactivity between the MAA and citrulline distinct epitope binding in RA [25]. Furthermore, unlike the high lung levels of MAA adducted protein observed in alcohol and cigarette smoke co-exposure [26], the lower concentration of ODE-induced MAA adducted protein would suggest that MAA represents an outcome, but not a cause of lung inflammation. Mechanisms underpinning the reduction in citrullinated and malondialdehyde-modified proteins in the lung of basal and ODE treated BCR KO mice are not understood and while this might be partly explained by the overall reduction in inflammatory mediators, lack of B cells, and reduction in exudative macrophages, further studies are necessary. It has been shown that MAA and citrulline co-localize in inflamed synovial tissues of RA patients with CD27 B cells [52]. Nonetheless, our study supports that repetitive ODE exposure-induced lung inflammation is associated with increased citrullinated and MAA modified proteins in the lung tissue with a corresponding serum ACPA and anti-MAA response.

There are limitations in this study. We do not know which lung inflammation-induced proteins underwent citrullination and/or MAA modification; it is reasonable to suspect that there are many potential candidates, one of which may be surfactant protein [26]. It is also possible that other autoantibodies could be induced by ODE-mediated lung jury including proteins modified by carbamylation and lipid oxidation. We have not identified specific anti-CIT or anti-MAA B cell clones, but others have described that B1 and memory B cells can produce these autoreactive antibodies [51]. B cell recruitment, maturation and activation can involve interaction with macrophages, T cells, resident B cells, and numerous mediators including, but not limited to IL-17A, IL-6, CXCL13, CXCL12, B cell activating factor (BAFF), a proliferating inducing ligand (APRIL) [34], and extracellular matrix proteins such as hyaluronan [33, 35]. Prior work has demonstrated key roles for IL-17A, Th1/Th17 lymphocytes, IL-6, and macrophages in ODE-mediated inflammation [12, 13, 53], and in this study we demonstrated again that ODE induces IL-17, but also found increased total hyaluronan as potential mechanisms to explain B cell recruitment. However, future studies are warranted to further understand this and other processes in ODE-mediated B cell regulation. It is also not known whether inhalant ODE exposure is directly linked to arthritis manifestations, and this line of work could be investigated in the context of collagen-induced arthritis or other relevant animal models of RA. These future studies could provide insight into the lung-autoimmune disease burden associated with agriculture work.

## Conclusions

There appears to be a central role for B cells and autoantibody reactivity following inhalant agriculture exposures. B cells were found to be critical for the development of ODE-induced lymphoid aggregates, and ODE exposure expanded B2, B1, and memory B cells populations. There was also evidence of increased ACPA and anti-MAA antibodies associated with ODE lung inflammation expression of citrullinated and MAA modified proteins. Future investigations in agriculture-exposed workers are therefore needed to better understand the clinical significance of these responses.

## Additional file

Additional file 1: Figure S1. Merged confocal images of isotype control antibodies with all 3 channels on each lung tissue section from WT and BCR KO mice treated repetitively with saline or ODE. Zenon 405 labeled rabbit IgG (401 nm to 421 nm) as control for MAA staining, mouse IgM+ anti-mouse IgM Cy3 (550 nm to 570 nm) as control for CIT staining, and ALEXA FLUOR® 594 Conjugated Rabbit IgG as control for CD68 macrophage staining. (PPTX 350 kb)

## Abbreviations

ACPA: Anti-citrullinated protein antibody
BALF: Bronchoalveolar lavage fluid
BCR: B cell receptor
COPD: Chronic obstructive pulmonary disease
Ig: Immunoglobulin
MAA: Malondialdehyde-acetaldehyde
MDA: Malondialdehyde
ODE: Organic dust extract
RA: Rheumatoid arthritis
TLR: Toll-like receptor
