# Supplementary material for: A role for B cells in organic dust induced lung inflammation
Source: Respir Res. 2017 Dec 22;18:214. doi: 10.1186/s12931-017-0703-x (PMC5741951; doi:10.1186/s12931-017-0703-x)
Supplement: Additional file 1: Figure S1. — Merged confocal images of isotype control antibodies with all 3 channels on each lung tissue section from WT and BCR KO mice treated repetitively with saline or ODE. Zenon 405 labeled rabbit IgG (401 nm to 421 nm) as control for MAA staining, mouse IgM+ anti-mouse IgM Cy3 (550 nm to 570 nm) as control for CIT staining, and ALEXA FLUOR® 594 Conjugated Rabbit IgG as control for CD68 macrophage staining. (PPTX 350 kb) [file 12931_2017_703_MOESM1_ESM.pptx]

## Slide 1
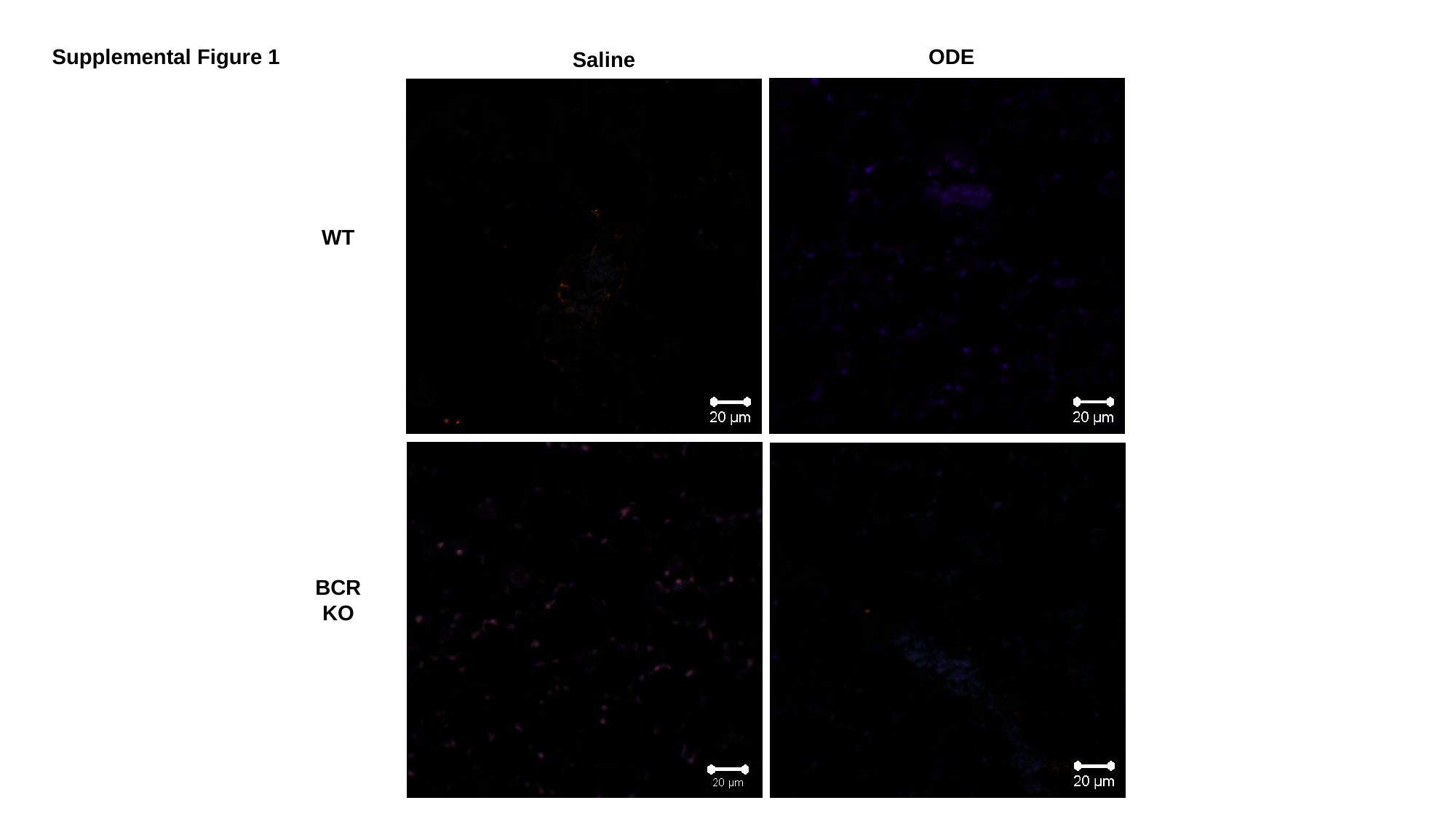

Supplemental Figure 1
ODE
Saline
WT
BCR KO

## Slide 2
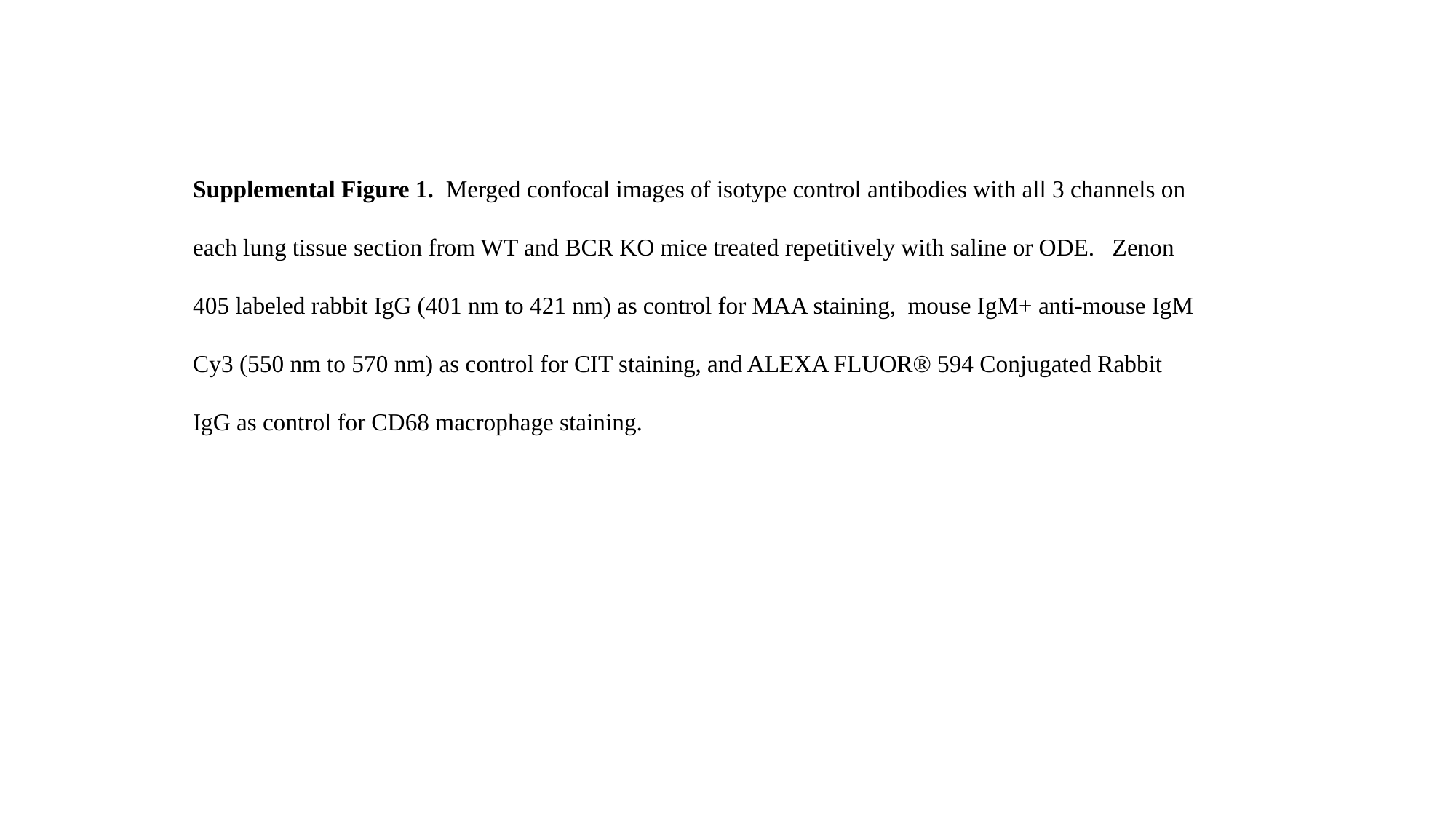

Supplemental Figure 1. Merged confocal images of isotype control antibodies with all 3 channels on each lung tissue section from WT and BCR KO mice treated repetitively with saline or ODE. Zenon 405 labeled rabbit IgG (401 nm to 421 nm) as control for MAA staining, mouse IgM+ anti-mouse IgM Cy3 (550 nm to 570 nm) as control for CIT staining, and ALEXA FLUOR® 594 Conjugated Rabbit IgG as control for CD68 macrophage staining.
